# Regulation of Neurological Devices and Neurointerventional Endovascular Approaches for Acute Ischemic Stroke

**DOI:** 10.3389/fneur.2018.00320

**Published:** 2018-06-11

**Authors:** Christopher M. Loftus, Michael Hoffmann, William Heetderks, Xiaolin Zheng, Carlos Peña

**Affiliations:** Division of Neurological and Physical Medicine Devices (DNPMD), Center for Devices and Radiological Health (CDRH), United States Food and Drug Administration (FDA), Silver Spring, MD, United States

**Keywords:** Food and Drug Administration, stroke, regulation, devices, endovascular

## Abstract

The United States Food and Drug Administration (FDA) Center for Devices and Radiological Health (CDRH) is charged with ensuring patients in the US have timely access to high-quality, safe, and effective medical devices of public health importance. Within CDRH, the Division of Neurological and Physical Medicine Devices reviews medical technologies that interface with the central and peripheral nervous system (neurotechnologies), including neurointerventional medical devices that are used in the treatment of stroke. Endovascular treatments have demonstrated recent advances in reaching the marketplace and providing more options for patients with acute ischemic stroke and intracranial aneurysms specifically. Depending upon the pathway chosen for regulatory approval, and the evidentiary standard for different regulatory pathways, neurotechnologies can have well-established safety and effectiveness profiles, varying degrees of scientific and clinical uncertainty regarding safety and effectiveness, or when a humanitarian use exists, need only demonstrate a probable benefit and safety to the patient so potentially life-saving treatments can reach the marketplace. Reperfusion therapies have had specific advances in the treatment of stroke patients that originally had limited or no treatment options and for preventative treatments in providing care to patients with intracranial aneurysms to avoid potentially more catastrophic outcomes. Collaboration in multiple forums and environments will be important to continue to foster the neurointerventional technology sector and positively impact clinical medicine, from diagnosing and treating a neurological disorder, to potentially altering the progression of disease, and in many ways, contemporary approved devices have brought a new sense of hope and optimism that serious and otherwise disabling neurological diseases can be treated and in many cases cured with modern therapy. We present here the scope of FDA’s regulatory landscape for neurological devices and neurointerventional endovascular approaches for acute ischemic stroke; this is essential information for those seeking to successfully translate medical device neurotechnologies for patient and consumer use.

Medical devices can have a significant impact on US patients, caregivers, and the overall health-care burden in the US, if safe and effective devices succeed in reaching the marketplace in a timely manner. Medical device technologies that interface with the central and peripheral nervous system (neurotechnologies) are an emerging area of rapid development, growth, and promise. Neurotechnologies have the potential for substantial public health impact on mental or physical impairments due to the nature of conditions being treated, such as stroke, epilepsy, Parkinson’s disease, Alzheimer’s disease, traumatic brain injury (TBI), spinal cord injury, brain tumors, and pain. Neurotechnologies span from diagnostics to preventative therapies to symptomatic treatments. Within the Food and Drug Administration (FDA), the vision of the Center for Devices and Radiological Health (CDRH) is to enable increasing access to safe and effective medical devices to US patients first in the world (1). In recognition of these priorities, the Division of Neurological and Physical Medicine Devices (DNPMD) serves as the primary point of review at the FDA for regulatory submissions involving medical device neurotechnologies. This article presents an overview of medical device regulation of neurotechnologies, a summary of CDRH’s review of neurotechnologies with an emphasis upon endovascular approaches for acute ischemic stroke, and information on how sponsors (companies or principal investigators who submit marketing applications or clinical investigations to the FDA) can best engage CDRH.

## The Framework for Regulatory Oversight of Neurotechnologies

The level of regulatory control for an individual device is based on a risk-based approach. CDRH determines the level of regulatory controls necessary for a medical device based upon its risks (i.e., probable harms or discomforts to a patient or user) (2), and classifies devices (in increasing risk) as Class I, II, or III, with regulatory control needed to provide reasonable assurance of safety and effectiveness increasing commensurately (3), and utilizing regulatory tools known as general and special controls. General controls apply to all devices and include good manufacturing practices, reporting of adverse events, device registration and listing with FDA, labeling, adulteration, and misbranding (3), while special controls are device-specific and include performance standards, such as post-market surveillance patient registries, special labeling requirements, premarket data requirements and guidelines. The device classification requirements are found in the Code of Federal Regulations (CFR) Title 21 for a general device type. Classification of neurological devices are outlined in 21 CFR Part 882 and physical medicine devices are outlined in 21 CFR Part 890 (4).

### Medical Devices Are Classified Based on Risk

There are several regulatory pathways available to bring medical devices to market. Most Class I devices, which require only general controls (e.g., good manufacturing practices, reporting of adverse events, device registration and listing with FDA) (3) to provide reasonable assurance of safety and effectiveness, are exempt from submitting an application to FDA; most Class II devices require Premarket Notification, also known as a 510(k) submission; and most Class III devices are subject to Premarket Approval (PMA). Alternatively, *De Novo* classification may be an appropriate regulatory pathway for class I or class II classification for medical devices for which general controls or general and special controls provide a reasonable assurance of safety and effectiveness, but for which there is no legally marketed predicate device. CDRH determines whether PMA and *De Novo* applications provide a “reasonable assurance of safety and effectiveness” by assessing if the probable benefits to health through the use of the device outweigh the probable risks of injury or illness from such use, while also considering other relevant factors. It is important to understand that Class II devices, in order to be cleared through the 510(k) pathway, require a comparable legally marketed Class II device (known as a “predicate device”) to which a sponsor can demonstrate “substantial equivalence” to the predicate device, meaning that the device has the same intended use as the predicate device and has either

(a) the same technological characteristics or
(b) has different technological characteristics and the information submitted, including appropriate clinical or scientific data, demonstrates that the device is as safe and effective as a legally marketed device, and does not raise different questions of safety and effectiveness than the predicate device (5).

### Safety and Effectiveness Data May Be Obtained Under an Investigational Device Exemption (IDE)

Center for Devices and Radiological Health is tasked to protect and promote public health, and it does so through the regulation of the design, manufacture, repackaging, relabeling, and/or the importing of medical devices into the US. Separate from the classification process described above specifically for marketing medical devices, CDRH also regulates medical device clinical investigations for investigational devices. However, many clinical investigations may proceed with approval from an IRB unless those investigations are deemed to be of significant risk to patients (i.e., present a potential for serious risk to the health, safety, or welfare of a subject) (6). Significant risk devices may include implants, devices that support or sustain human life, and devices that are substantially important in diagnosing, curing, mitigating or treating disease or in preventing impairment to human health. Under these circumstances, sponsors must obtain FDA approval of an IDE prior to patient enrollment when studies are conducted in the US. Data collected from these studies may later be used in support of additional studies or marketing applications. In 2011, the median time to full approval of an IDE submission was more than 400 days. Following the implementation of the Food and Drug Act Safety and Innovation Act and through the increased interaction of FDA and sponsors during review, the time to full approval of an IDE study has been dramatically reduced since 2013. As of 2016, CDRH has had an overall median time to approval of 30 days (7). Sustaining these rapid review times of IDE submissions remains a priority for DNPMD, as the acceleration of device development and research is critical to the realization of research investments and getting safe and effective devices to the public.

### Medical Device and Drug Combination Products

There are exigencies where neurological devices are used with or to deliver drugs or biologics to targeted areas of the nervous system. In such situations, where the scope of authority may overlap between centers at FDA, DNPMD also works with its medical product counterparts in the Center for Drug Evaluation and Research and Center for Biologics Evaluation and Research to evaluate aspects of the proposed device/drug/biologics products. Typically, the lead Center conclaves with the associated product Centers to meet, discuss, and recommend an approach to study the combination product in a clinical trial, or to render a decision regarding a marketing application.

## Oversight of Neurointerventional Devices Related to Stroke

Neurointerventional devices have been the focus of attention recently, with rapid evolution and advancement of treatments, such as mechanical neurothrombectomy devices for stroke and endovascular devices to treat intracranial aneurysms. For any medical device to reach the marketplace, it helps for sponsors to be familiar with the existing regulatory framework and understand the regulatory pathways available for their device. CDRH reviews each medical device submission along a regulatory path that is in part tailored to address: (1) the individual risk of the device to the patient and (2) the least burdensome pathway to market. This means that CDRH must carefully consider and apply adequate oversight for any given device, while at the same time, enable a regulatory framework that is flexible to be responsive to the development of new and innovative technologies.

Currently, there are several devices with market authorization for preventing and treating stroke. For example, FDA has approved two intracranial stent systems for the treatment of symptomatic and medically refractory intracranial atherosclerotic disease: the Wingspan Stent System (Boston Scientific, 2005) and NeuroLink System (Guidant, 2002). FDA has approved the Pipeline Embolization Device, a flow diverter, for the treatment of large and giant wide-neck intracranial aneurysms in certain locations of the neurovasculature. In 2004, the first mechanical thrombectomy device (the Merci Retriever) was cleared through the premarket notification [510(k)] pathway to restore blood flow in the neurovasculature by removing thrombus in patients experiencing an acute ischemic stroke who were ineligible to receive intravenous tissue plasminogen activator (IV t-PA), or who failed IV t-PA therapy. This initial clearance led to the subsequent clearances of additional neurothrombectomy devices with stent-retriever like designs such as the Solitaire Revascularization Device (Micro Therapeutics, Inc.,d/b/a ev3 Neurovascular, 2012), Trevo Retrievers (Concentric Medical, Inc., 2012), and the MindFrame Capture LP Revascularization Device (Micro Therapeutics, Inc., d/b/a ev3 Neurovascular, 2015). In addition, aspiration catheters with vacuum pump designs such as the Penumbra System (Penumbra, Inc., 2007) and the Riptide Aspiration System (Micro Therapeutics, Inc., d/b/a ev3 Neurovascular, 2017) were developed and cleared for an indication for use in patients similar to the patient population of the original Merci Retriever clearance, that is: revascularization in acute ischemic stroke patients who are ineligible to receive or failed IV t-PA therapy.

In 2016, the FDA created a new regulation (21 CFR 882.5600, Neurovascular Mechanical Thrombectomy Device for Acute Ischemic Stroke Treatment, product code POL) and associated special controls, with the granting of a *De Novo* request for the Trevo Retrievers (Concentric Medical, Inc.), “for use to restore blood flow in the neurovasculature by removing thrombus for the treatment of acute ischemic stroke to reduce disability in patients with a persistent, proximal anterior circulation, large vessel occlusion, and smaller core infarcts who have first received intravenous tissue plasminogen activator (IV t-PA).” As stated in the product labeling, endovascular therapy with the device should start within 6 h of symptom onset.

Also in 2016, the Solitaire Revascularization Device (Micro Therapeutics, Inc., d/b/a ev3 Neurovascular) was cleared with this same treatment indication—of reduction in disability in acute ischemic stroke patients. The two studies used to support the treatment indication were both randomized controlled trials (RCTs), and they are two of five published RCTs (i.e., MR CLEAN, SWIFT-PRIME, EXTEND-IA, ESCAPE, REVASCAT) that were made public in the same time frame (8–12). These published RCT studies supported the safety and effectiveness of these two devices (e.g., stent-retrievers) in reducing disability in certain acute ischemic stroke patients if treated within 6 h from stroke symptom onset.

Regardless of the submission pathway, weighing the benefits and risks is an essential part of CDRH’s determination of whether a medical device is ready for the US marketplace. For example, for its highest risk devices, CDRH determines whether PMA and *De Novo* applications provide a “reasonable assurance of safety and effectiveness” by “weighing any probable benefit to health through the use of the device against any probable risk of injury or illness from such use,” among other relevant factors (13). Once data supporting the safety and effectiveness (or substantial equivalence, as appropriate) of certain medical devices has been collected, sponsors apply to CDRH to legally market their device in the US. When the longer-term risks of a medical device are not yet completely understood, yet the safety and effectiveness support market approval, patient access to the device may be granted, with the agreement for additional long-term data, in some cases collected from a post-approval study. CDRH has published guidance to share its thinking on how a medical device with these types of uncertainty can continue to be monitored in the post-market arena (i.e., post-market study) (14).

## Engaging the FDA: Best Practices

### The Pre-Submission—An Opportunity to Open a Dialog With FDA

Public engagement is critical for the agency—to ensure that sponsors understand the regulatory requirements necessary to bring a product to the marketplace. Sponsors are encouraged to interact early and often with FDA staff, with the goal that harmonization of objectives and expectations between FDA and sponsors is best achieved at the earliest possible stages—ideally before any resources are expended. The Presubmission pathway is the primary method of engaging the agency, from informational meetings to more specific responses to sponsor questions. For example, a Pre-Submission application can be used to (1) evaluate the inclusion criteria, assessment tools, and study endpoints for a given study, (2) ascertain what should be the study design or specific data needed to support approval of a future marketing submission (15), and/or (3) to determine the type of IDE study to pursue (e.g., early feasibility study, traditional feasibility study, or pivotal study) for the development of a given medical device. Pre-Submissions may also be used to provide feedback on protocols or testing related to other premarket submission types [e.g., 510(k)s, *De Novo*].

### Guidance Documents—An Approach to Providing Sponsors With FDA Expectations

Center for Devices and Radiological Health publishes guidance documents for medical devices to outline information that may be most useful in a submission, including, for example, nonclinical and clinical data requirements. Since medical device regulations recognize many types of valid scientific evidence, including randomized clinical trials, well-controlled investigations, partially controlled studies, studies, and objective trials without matched controls, well-documented case histories conducted by qualified experts, and reports of significant human experience, the availability of guidance helps to provide advice on specific medical device types and technologies. Thus, the availability of specific guidance can serve as a vitally important resource for sponsors. For example, FDA released final guidance in November 2016 that focuses on conducting clinical trials for medical devices that target neurological disease progression (16); a revolutionary approach to treating disease, rather than addressing symptoms alone. This guidance outlines CDRH’s current thoughts about data necessary to support an IDE application—and to help in the design of clinical trials.

### Public Meetings and Workshops—Direct Communication With FDA Staff

Food and Drug Administration may decide that public discourse is a direct, practical, and timely way to discuss specific neurotechnologies, and this discourse can be implemented either by federally organized workshops and symposia, or by participating in forums and conferences organized by outside parties. There are several recent examples of this transparency on FDA’s part, including CDRH’s 2014 public workshop on Brain Computer Interfaces, which discussed challenges with device development, translation to human studies, and brainstorming of solutions, the 2015 Stroke Public meeting that assembled experts in the field of stroke to consider recent advances in the neurothrombectomy space as it relates to existing guidance and to galvanize communities to assess the state of the art in stroke (17), and CDRH’s 2016 public meeting on biomarkers and other assessments in TBI (18). More recently, in 2017, DNPMD staff participated in the HEmorrhagic stroke Academia inDuStry Roundtable (19). Last and most recent of all, DNPMD leadership presented at the 2017 STAIR X meeting, an industry/academic/FDA colloquium to share FDA’s perspective in bringing forth stroke trials and trial design issues to the stroke community. Overall, FDA’s public meetings aim to assess the current state of science, evaluate the clinical understanding and treatment of disease, foster the development of discourse for sponsors to aid in moving devices to patients and consumers, and increase the attention to existing regulatory requirements in place.

### Partnerships With Public and Private Stakeholders, Academic Leaders, and Industry

Food and Drug Administration has invested significant staffing and resources in partnerships not only within the agency but also through broader collaborations and activities with other government agencies and with public and private entities. One example of public–private partnership with both other government entities and the private sector is the Devices used for Acute Ischemic Stroke Intervention (DAISI) CRN initiative. DAISI is an FDA/organized medicine/industry sponsor partnership initiative to use vetted real-world registry datasets to inform specific regulatory decisions and IFU labeling for neurological devices.

Division of Neurological and Physical Medicine Devices has also created and hosted four webinars (on *De Novo*, Humanitarian Device Exemption, IDE, and PMA applications), to inform sponsors and other interested parties about the regulatory oversight and decision-making process.

Division of Neurological and Physical Medicine Devices staff regularly engage with several national and international stroke, neurology, and neurosurgery societies, in many and in flexible forms depending on the situation, and this targeted engagement is intended to foster transparency of the regulatory processes—and to allow in-person introductions of FDA processes to academic and industry principals. Overall, FDA’s public meetings aim to assess the current state of science, to evaluate the clinical understanding and treatment of disease, to foster the development of new guidance documents for sponsors to aid in moving devices to patients and consumers, and to increase the attention to existing regulatory requirements in place.

### Additional Resources Available to the Public

There exist additional resources which the public can access to further increase the transparency of the regulatory landscape for neurological devices. For example, the DNPMD maintains a neurological devices webpage online (20). The DNPMD has also published articles on its activities to foster increased understanding of FDA processes and provide a reference for sponsors, investigators, and other interested parties. For example, an invited review of DNPMD processes was published in Neuron in 2016 (21). This current invited submission is another example of this same initiative.

## Summary

Center for Devices and Radiological Health plays a key role in enabling access of high-quality, safe, and effective medical devices of public health importance, including neurological and physical medicine devices, to all Americans first in the world, and by indirect extension, to patients around the world. Determining the safety, effectiveness, benefits, and risks of a medical device involve an assessment of the data in terms of both the clinical (is there a clinically meaningful benefit?) and the statistical findings, while folding in patient experience perspectives when applicable. Early discussions with sponsors help to ensure transparency and clear expectations on the evidence needed to move a medical device into the marketplace. CDRH takes a least burdensome approach by tailoring our scientific and regulatory review to the specific profile of a medical device, making clear, transparent, and predictable scientific-based decisions, to ensure that CDRH is meeting the public’s expectations of government institutions and its mission of protecting and promoting the public health.

## Author Contributions

All authors listed have made a substantial, direct, and intellectual contribution to the work and approved it for publication.

## Conflict of Interest Statement

The authors declare that the research was conducted in the absence of any commercial or financial relationships that could be construed as a potential conflict of interest.
